# Evaluation of PACE4 isoforms as biomarkers in thyroid cancer

**DOI:** 10.1186/s40463-018-0311-x

**Published:** 2018-10-19

**Authors:** Laurent Fradet, Rabia Temmar, Frédéric Couture, Mathieu Belzile, Pierre-Hugues Fortier, Robert Day

**Affiliations:** 1 Division of Otolaryngology, Department of Surgery, Faculty of Medicine, Université de Sherbrooke, CIUSSS de l’Estrie – CHUS, Hôpital Hôtel-Dieu de Sherbrooke, 580 Bowen S, Sherbrooke, QC J1G 2E8 Canada; 2 Department of Pathology, Faculty of Medicine, Université de Sherbrooke, CIUSSS de l’Estrie – CHUS, Hôpital Hôtel-Dieu de Sherbrooke, 580 Bowen S, Sherbrooke, QC J1G 2E8 Canada; 3 Divison of Urology, Departemnt of Surgery, Faculty of Medicine, Université de Sherbrooke, Institut de pharmacologie de Sherbrooke, 3001 12th Ave N, Sherbrooke, QC J1H 5N4 Canada

**Keywords:** Molecular marker, Biomarker, Thyroid nodule, Thyroid Cancer, Fine needle aspiration, Proprotein convertase PACE4

## Abstract

**Background:**

To date, no single molecular marker has been demonstrated as clinically useful in differentiating malignant from benign thyroid nodules when a fine needle aspiration falls in the “unknown significance” categories of the *Bethesda Classification*. PACE4, a member of the proprotein convertase family of enzymes, has been shown to play a major role in the pathogenesis of prostate cancer, through the formation of an oncogenic isoform named PACE4-altCT. PACE4 isoforms have also been suggested to play a role in other cancers, including thyroid cancer, but have never been investigated in a detailed manner. Our objective is to compare the histochemical distribution of the two major PACE4 isoforms in benign and malignant thyroid nodules, in order to determine their potential usefulness as discriminatory biomarkers.

**Methods:**

Thyroid tissues of patients who underwent thyroidectomy were classified according to final pathology. Corresponding tissue sections were immunostained, using two previously validated antibodies raised against the C-terminal end of the two PACE4 isoforms, namely the full-length PACE4 protein (PACE4-FL) and its alternative isoform (PACE4-altCT). Nodules were compared with adjacent normal parenchyma and immunostaining was rated as “low” or “high” by a head and neck pathologist.

**Results:**

Non-lesional thyroid parenchyma did not express PACE4-FL (*p* = 0.002). As a group, malignant (*n* = 17) nodules expressed PACE4-FL significantly more than benign (*n* = 24) nodules (percentage of high immunostaining: 52.9% vs 4.2%; *p* = 0.001). Reciprocally, there was a statistically lower expression of PACE4-altCT in malignant nodules than in adjacent non-lesional parenchyma (*p* = 0.014). The specificity of a high PACE4-FL immunostaining in determining malignancy was 95.8% (95% CI, 78.9% to 99.9%).

**Conclusion:**

This study supports the previously described relationship between PACE4-FL and PACE4-altCT through alternative splicing. It also suggests that PACE4-FL is a promising biomarker for thyroid malignancy. Its high specific expression for malignancy could make it an interesting “rule in” test for thyroid cancer. Further prospective, quantitative studies are currently being designed to address how measurements of PACE4 isoforms could be used in a clinical setting.

**Trial registration:**

This study does not report the results of a health care intervention on human participants. It was nonetheless registered on *ClinicalTrials.gov* under reference number NCT03160482.

**Electronic supplementary material:**

The online version of this article (10.1186/s40463-018-0311-x) contains supplementary material, which is available to authorized users.

## Background

It is estimated that up to 68% of the general population present at least one detectable thyroid nodule on ultrasound [1]. Current investigation guidelines, published by the *American Thyroid Association* in 2015, are based on sonographic characterisation of the nodules, followed by fine needle aspiration, depending on their size and features. Cytopathology results are then reported according to the *Bethesda Classification*, providing a diagnostic category, along with its estimated risk of malignancy. However, only 55 to 74% of analyzed nodules will be interpreted by cytopathologists as definitively benign and 2 to 5% as definitively malignant [2]. Remaining nodules will fall in a category of “unknown significance”: 2 to 18% of samples will be categorized as “Atypia of unknown significance – Follicular lesion of unknown significance” (AUS/FLUS; risk of malignancy, 6–18% [3]), 2 to 25% as “Follicular neoplasm – Suspicion of follicular neoplasm” (FN/SFN; risk of malignancy, 10–40% [3]), and 1 to 6% as “Suspicious for malignancy” (SUSP; risk of malignancy, 45–60% [3]) [2]. A majority of these patients will end up being operated, despite the relatively low malignancy risk of these categories [4]. The cost of a total thyroidectomy is estimated to about 6 000 USD [5], and the potential morbidity related to this surgery is significant [6].

In recent years, researchers have been working on molecular markers that could help to differentiate malignant from benign thyroid nodules when a fine needle aspiration falls in these “unknown significance” categories. However, as stated by the *American Thyroid Association*, “there is currently no single optimal molecular test that can definitively rule in or rule out malignancy in all cases of indeterminate cytology, and long-term outcome data proving clinical utility [of commercialized biomarkers] are needed” [2]. This justifies the search for new biomarkers.

Paired amino acids converting enzyme 4 (PACE4) is a member of the proprotein convertase family of enzymes. A growing number of publications highlight the role of this protease in carcinogenesis and tumor progression [7–9]. PACE4 plays a role in the oncogenesis of prostate, ovary, and breast cancer [10–12]. More recently, two PACE4 isoforms have been described by Couture et al.: a full-length isoform, PACE4-FL, and its alternative isoform, PACE4-altCT, which is different from its parent isoform in term of autocatalytic processing and cellular trafficking, as it remains inside the secretory pathway without being secreted. The difference between these two isoforms results from an alternative splicing of the PACE4 transcript [13]. In prostate cancer, the PACE4-altCT isoform was shown to be oncogenic. In addition, preliminary data also suggest that this splicing event can also be found in other tissues, including thyroid, and thus could be involved in cancer related events [13].

The objective of this study is to describe the expression of the two PACE4 isoforms among benign and malignant thyroid nodules, in order to determine their value as potential molecular markers for thyroid cancer.

## Methods

### Study design

This pilot study was designed as a transversal, descriptive study. Our goal is to describe the expression profile of PACE4 isoforms among thyroid nodules and adjacent normal parenchyma in order to justify further prospective, quantitative studies on this proprotein, that could lead to clinical applications.

### Ethics approval

This research protocol was presented to the Institution Ethic Board (*CIUSSS de l’Estrie – CHUS*; certified FWA #00005894 and IRB00003849) and granted with full approval.

### Patients samples

A list of all patients who underwent either total thyroidectomy or thyroid lobectomy at the *CIUSSS de l’Estrie – CHUS*, from January 2014 to May 2016, was prepared by the medical archives.

**Fig. 1:**
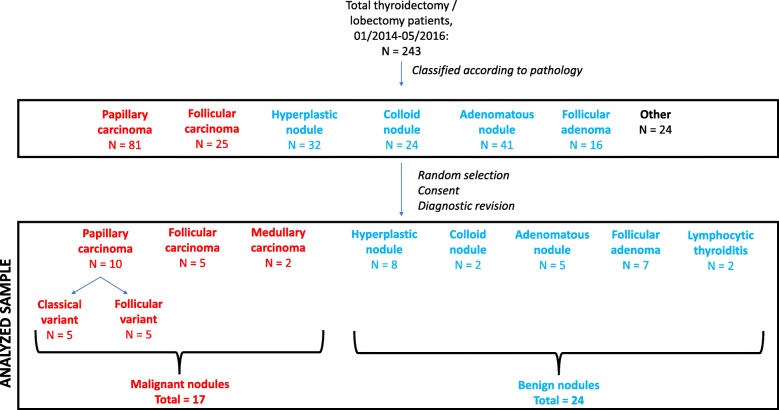
Patient flowchart

These 243 medical files were reviewed by the main investigator (Fig. 1). Patients were classified based on final pathological diagnosis. We chose to study the most common variants of well-differentiated thyroid cancer, i.e. papillary carcinoma (specifically, both its classical and follicular variants) and follicular carcinoma. For benign nodules, we also chose to study the most frequently encountered types, i.e. hyperplasic, colloid and adenomatous nodules, and follicular adenoma. Recruitment was continued randomly until a total of 5 patients per diagnostic category was reached. We estimated that this number of patients per category would be sufficient to observe trends in results. We added to this cohort two cases of medullary cancer that were identified in our database and two cases of lymphocytic thyroiditis, along with two more cases of follicular adenoma that were previously classified as “oncocytic nodules”, for a total of 41 patients (Fig. 1). Revision of the final pathology led to three colloid nodules being reclassified as hyperplastic nodules. As such, only two sample were analysed for the colloid nodule category and eight fell in the hyperplastic nodule category (Fig. 1).

Patients were contacted by the main investigator (LF) for consent. Upon consent, pathological slides of each patient were reviewed by a head and neck pathologist (RT) to confirm the diagnosis. In particular, follicular variants of papillary carcinoma were thoroughly reviewed to make sure they were not reclassified as the newly described variant of *Non-invasive Follicular Thyroid Neoplasm with Papillary-like Nuclear Features* (NIFTP), which is considered a benign neoplasm. New 4 μm-thick slides were cut from paraffin blocs and were used to perform the immunohistochemistry technique. Blocks were specifically chosen by the pathologist to ensure that both lesional (nodule) and non-lesional (adjacent normal) thyroid parenchyma would be found on each slide, in order to compare the expression of PACE4 in these two components.

### Immunohistochemistry technique

Rabbit polyclonal immunoglobulins targeting either PACE4-FL or PACE4-altCT were purified on an antigenic peptide-coated chromatographic column. The sensitivity and specificity of these antibodies was previously demonstrated [13].

Using these antibodies, an automatized immunohistochemistry technique was performed with a Dako device (Agilent technologies, Santa Clara, CA). Slides were stained with a horseradish-peroxidase reaction and counterstained with Harris hematoxylin (Sigma-Aldrich, St-Louis, Missouri). Detailed description of this technique was previously published by Couture et al. [13].

Before performing this technique on our recruited patient sample, validation of the immunohistochemistry method was undertaken using four slides of thyroid tissues that included both non-lesional parenchyma and specific types of nodules. This enabled us to confirm that the thyroid tissue stained properly with this technique.

### Data acquisition

Immunostained slides were interpreted by a specialized head and neck pathologist (RT). The intensity of immunostaining was described as either “low” (for no to slight staining) or “high” (for moderate to intense staining), both for the studied nodule (i.e. lesional parenchyma, either cancerous or benign) and for adjacent non-lesional (i.e. normal) parenchyma. Captions of representative fields were taken with a microscope digital camera (Olympus DP26; Olympus, Tokyo, Japan), under 20X magnification.

### Statistical analysis

Statistical analyses were independently run and validated by the institution biostatistician.

Wilson’s 95% confidence intervals were calculated on the percentage of high immunostaining for every diagnosis.

Comparison of the percentage of high immunostaining between lesional and non-lesional parenchyma, for each antibody, was made with the McNemar test on two by two contingence tables using SAS Software, version 9.3 (SAS Corporation, Cary, NC).

Comparison of the percentage of high immunostaining between cancerous and benign nodules, for each antibody, was made with the Fischer exact test on two by two contingence tables using SPSS Software, version 25 (IBM, Armonk, NY). Results from multiples comparisons between each pair of diagnosis were obtained by Fisher exact tests and *p*-values were corrected by the false discovery rate method, as described by Benjamini and Hochberg.

The sensitivity and specificity of each PACE4 isoform, with 95% confidence intervals, were determined on two by two contingence tables using the MedCalc online software (www.medcalc.com).

## Results

### Data spreadsheet

The original data spreadsheet is available as Additional file 1.

### Expression of PACE4-FL

**Fig. 2:**
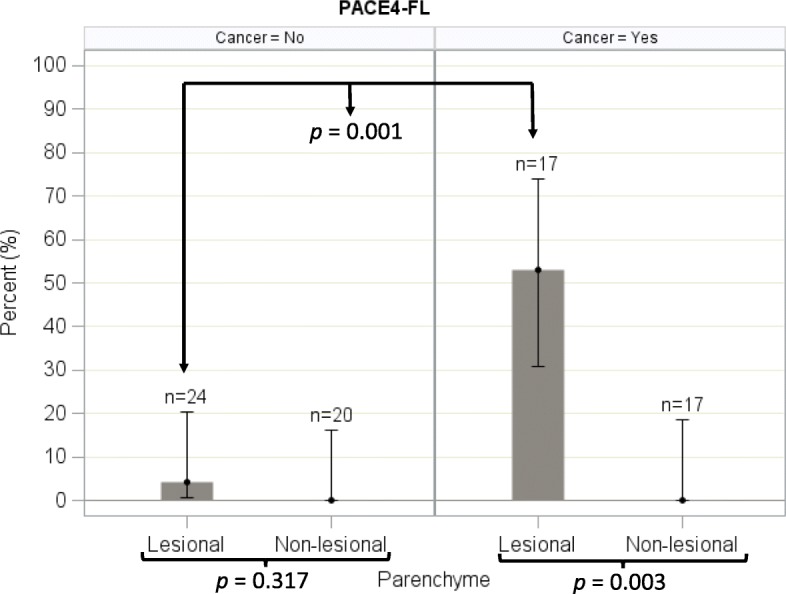
Proportion of high immunostaining in lesional (nodule) and non-lesional (normal) thyroid parenchyma, PACE4-FL. Error bars represent the Wilson’s 95% confidence intervals

**Fig. 3:**
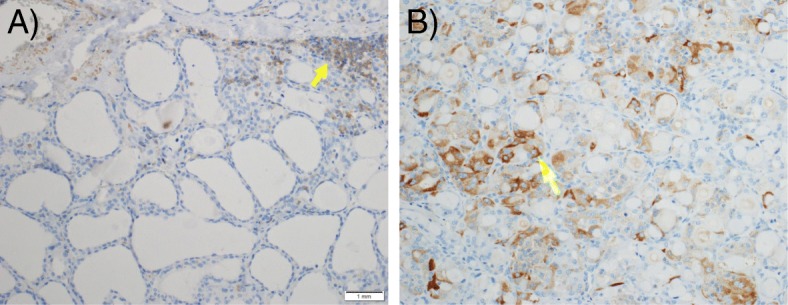
Comparison of representative fields for a case of follicular carcinoma (malignancy), PACE4-FL, under 20X magnification. **a** Non-lesional (normal) parenchyma, which did not exhibit staining; the only immunostained cells are lymphocytes (arrow). **b** Lesional parenchyma (nodule), demonstrating high immunostaining (arrow)

For all of the 37 samples in which non-lesional parenchyma was identified, a low PACE4-FL immunostaining was observed (Fig. 2). There was no statistical difference between the expression of PACE4-FL in lesional and non-lesional parenchyma for benign lesions, which both exhibited a low expression of PACE4-FL (*p* = 0.317; Fig. 2). However, the expression of PACE4-FL was statistically higher in lesional than in non-lesional parenchyma for malignant lesions (*p* = 0.003; Figs. 2 and 3).

**Fig. 4:**
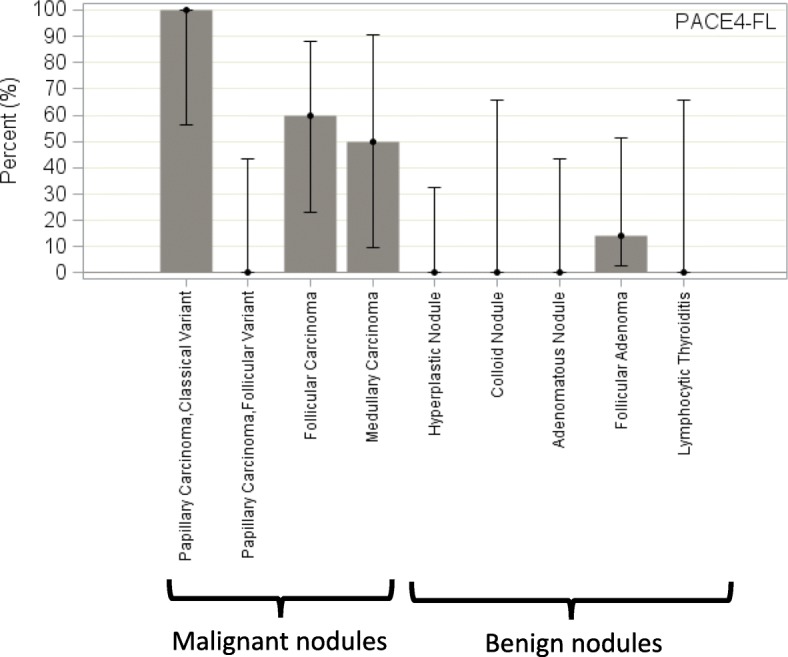
Percentage of high immunostaining for every nodule type (lesional parenchyma), PACE4-FL. Error bars represent the Wilson’s 95% confidence intervals

**Fig. 5:**
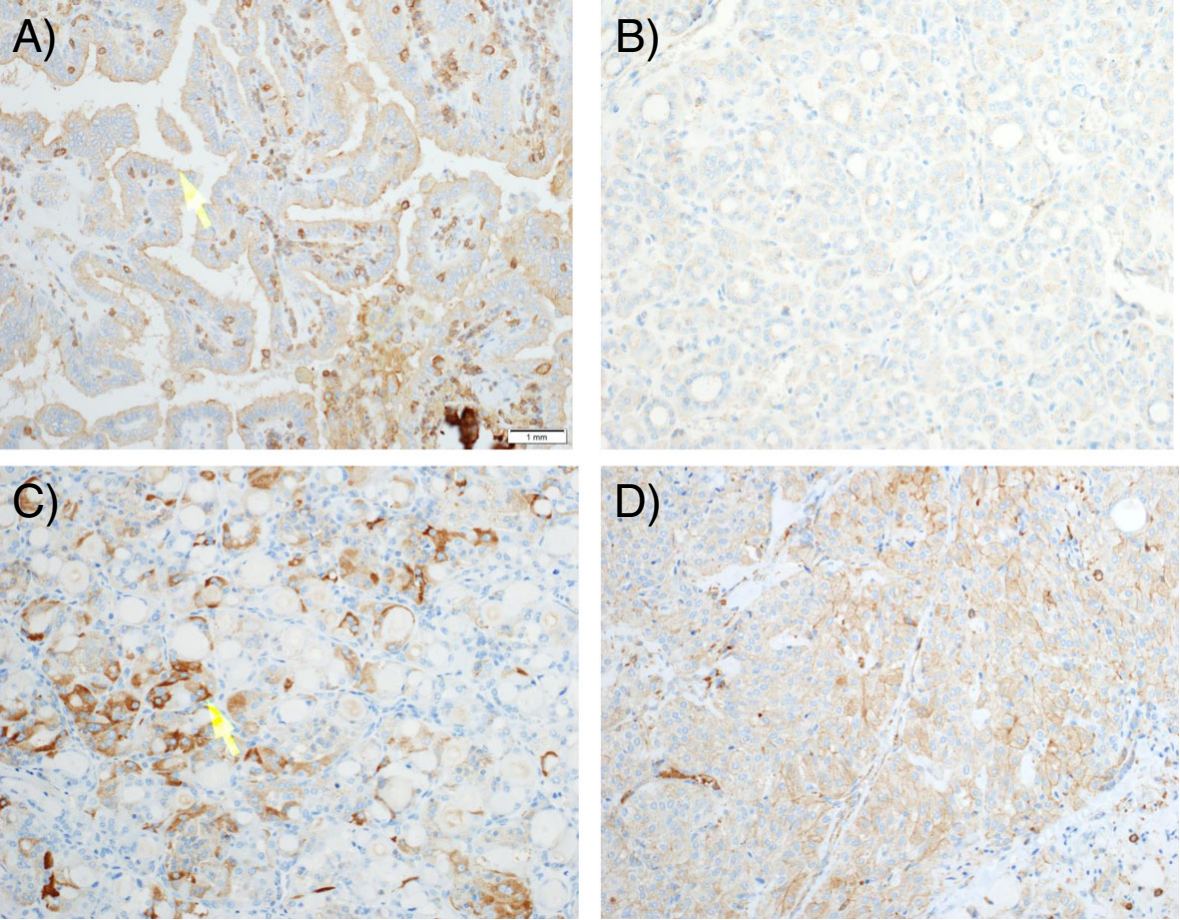
Representative fields for malignant nodules, PACE4-FL, magnification 20X. **a** Papillary carcinoma, classical variant (high immunostaining; arrow). **b** Papillary carcinoma, follicular variant (low immunostaining). **c** Follicular carcinoma (high immunostaining; arrow). **d** Medullary carcinoma (high immunostaining)

**Fig. 6:**
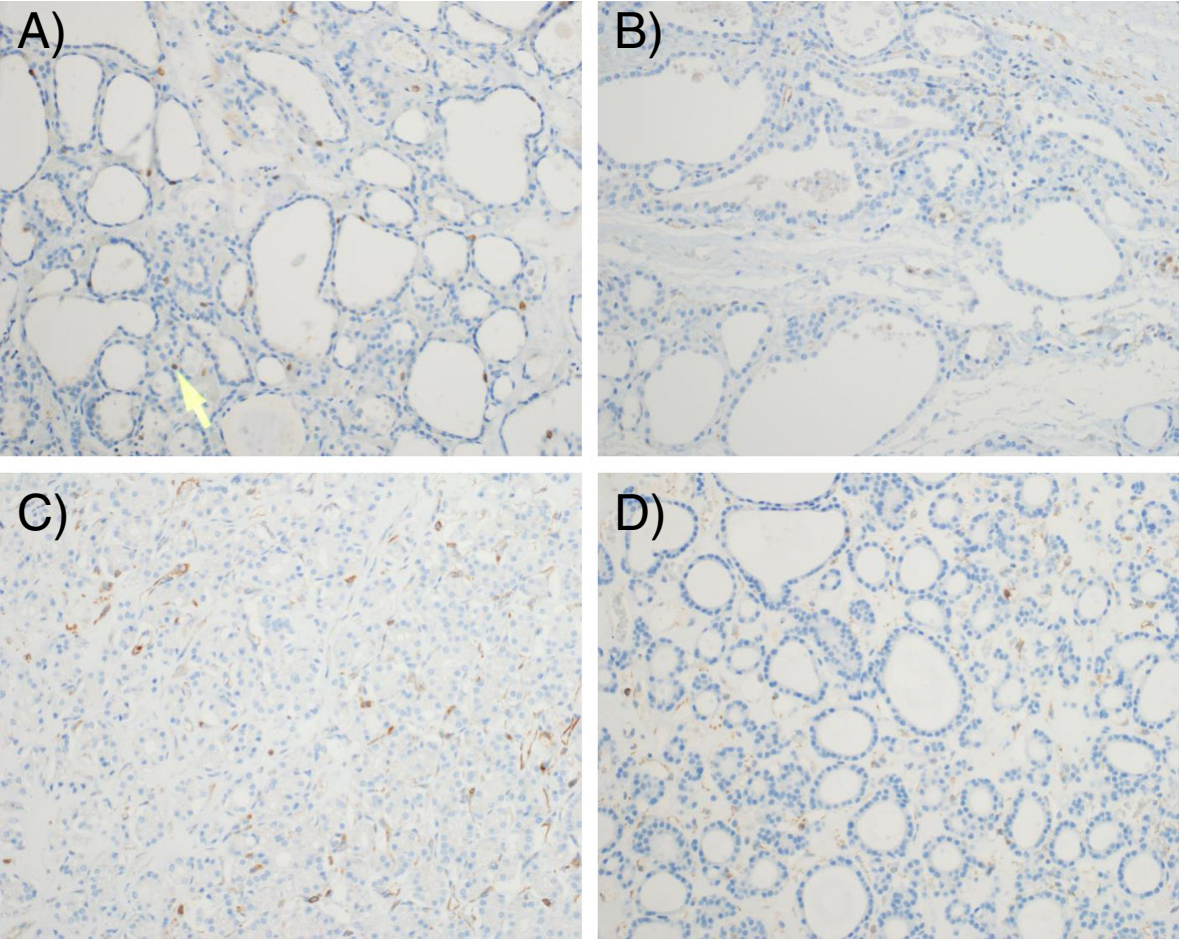
Representative fields for benign nodules, PACE4-FL, magnification 20X. **a** Hyperplastic nodule; the arrow indicates an immunostaining lymphocyte. **b** Colloid nodule. **c** Adenomatous nodule. **d** Follicular adenoma

As a group, malignant nodules expressed PACE4-FL to levels significantly higher than benign nodules (percentage of high immunostaining: 52.9% vs 4.2%; *p* = 0.001; Fig. 2). Multiple comparison analyses of the proportion of high immunostaining among the different nodule types can be found in Additional file 2. The sensitivity and specificity of a high PACE4-FL immunostaining in determining malignancy were respectively 52.9% (95% CI, 27.81% to 77.02%) and 95.8% (95% CI, 78.9% to 99.9%). Figure 4 presents the percentage of high immunostaining for every nodule type; representative fields are found in Fig. 5 for malignant and in Fig. 6 for benign nodules.

In malignancies, expression of the protein was most pronounced for the classical variant of papillary carcinoma, which displayed an apically-distributed high immunostaining in all five studied samples (Fig. 5a). On the opposite, immunostaining was low for all follicular variant of papillary carcinoma specimens examined (Fig. 5b). For follicular carcinoma, three out of five slides displayed high immunostaining, which was especially pronounced in the more compact, oncocytic zones of the nodules (Fig. 5c, arrow). One medullary carcinoma included in this study showed a high immunostaining (Fig. 5d), while the second displayed a low immunostaining.

All benign nodule types consistently showed no expression of PACE4 (Figs. 2, 4 and 6), except for one follicular adenoma sample.

### Expression of PACE4-altCT

**Fig. 7:**
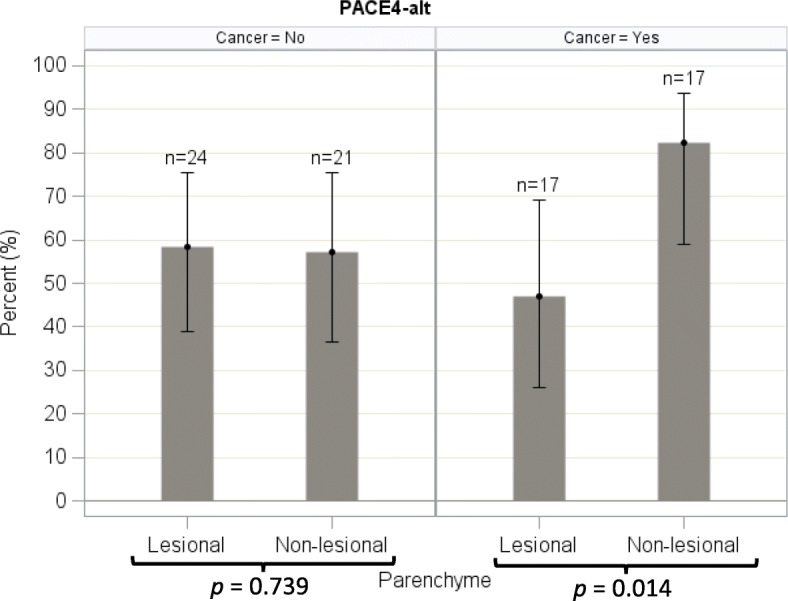
Proportion of high immunostaining in lesional (nodule) and non-lesional (normal) thyroid parenchyma, PACE4-altCT. Error bars represent the Wilson’s 95% confidence intervals

**Fig. 8:**
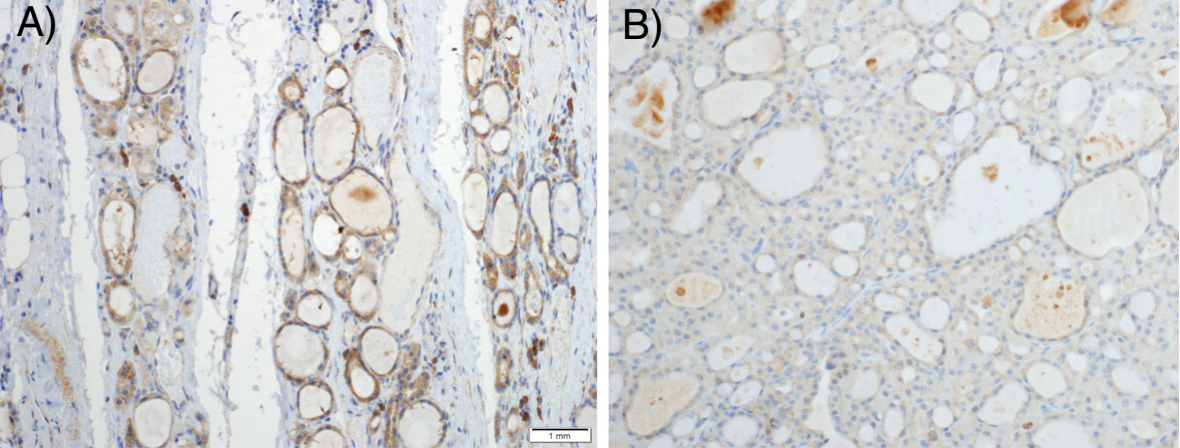
Comparison of representative fields for a case of follicular carcinoma (malignancy), PACE4-altCT, 20X magnification. **a** Non-lesional (normal) parenchyma (high immunostaining). **b** Lesional (nodule) parenchyma (low immunostaining)

For benign nodules, PACE4-altCT was equally expressed in both lesional and non-lesional thyroid parenchyma (*p* = 0.739; Fig. 7). However, for malignant nodules, there was a statistically higher expression of PACE4-altCT in non lesional parenchyma, compared to lesional parenchyma (*p* = 0.014; Figs. 7 and 8).

**Fig. 9:**
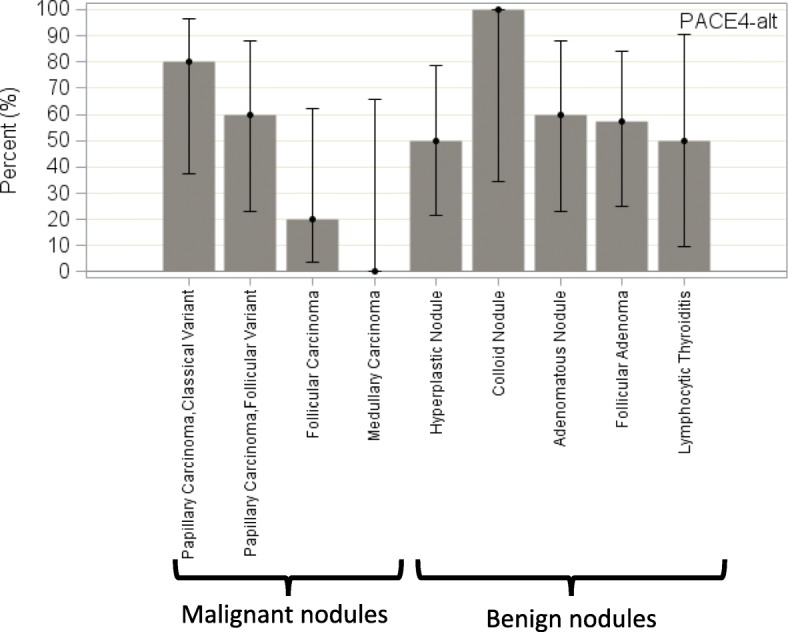
Percentage of high immunostaining among every nodule type (lesional parenchyma), PACE4-altCT. Error bars represent the Wilson’s 95% confidence intervals

**Fig. 10:**
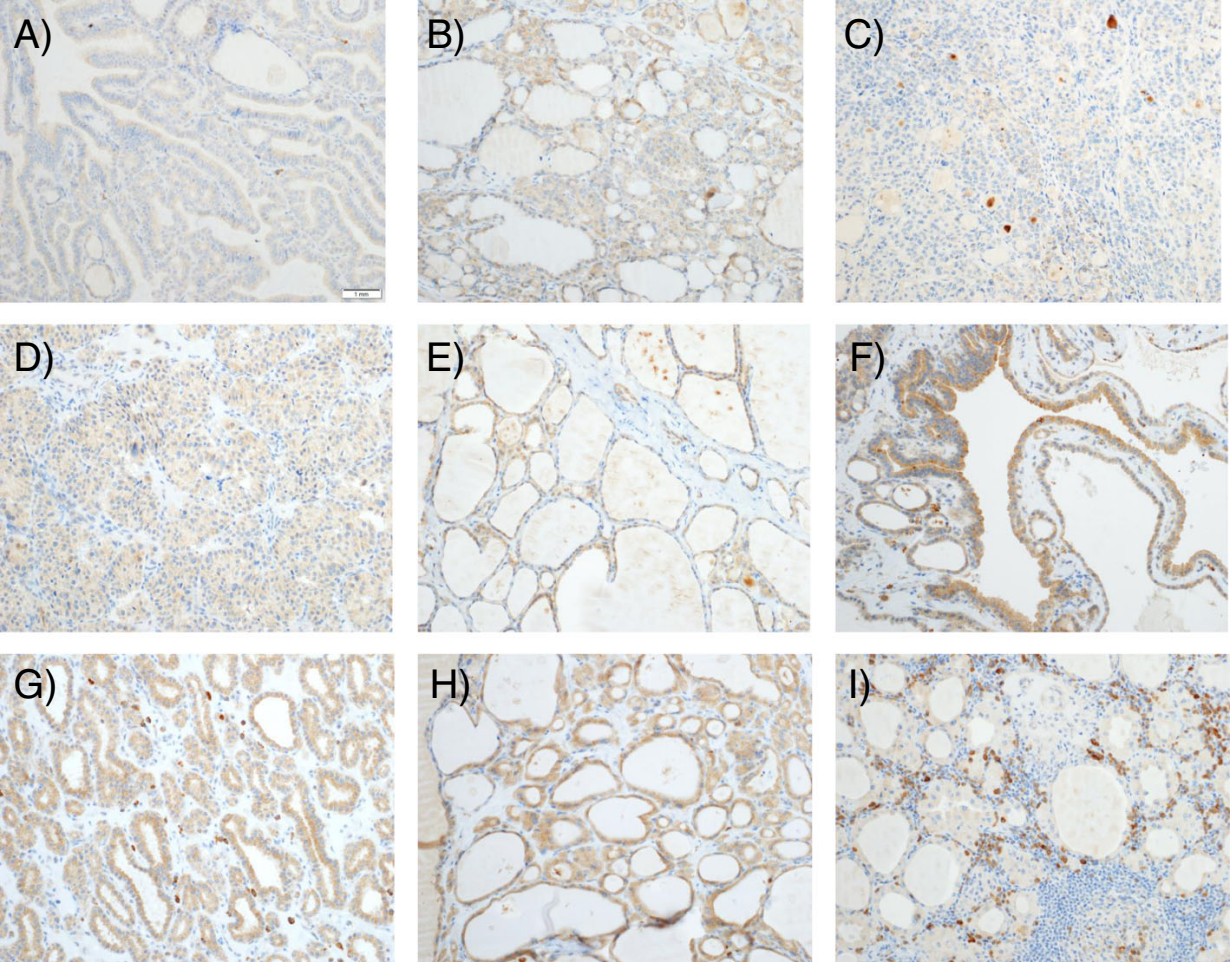
Representative fields, PACE4-altCT, 20X magnification. **a** Papillary carcinoma, classical variant. **b** Papillary carcinoma, follicular variant. **c** Follicular carcinoma. **d** Medullary carcinoma. **e** Hyperplastic nodule. **f** Colloid nodule. **g** Adenomatous nodule. **h** Follicular adenoma. **i** Lymphocytic thyroiditis

As a group, malignant nodules did not express PACE4-altCT significantly more than benign nodules (percentage of high immunostaining: 47.1% vs 58.2%; *p* = 0.537; Fig. 8). The sensitivity and specificity of PACE4-altCT for differentiating malignant and benign nodules, based on a high immunostaining, were respectively 47.1% (95% CI, 23.0% to 72.2%) and 41.7% (95% CI, 22.11% to 63.4%). When comparing the percentage of high immunostaining from one type of nodule to the other, no significant trend could be noted (Fig. 9; Additional file 2). While the heterogeneity of results make it difficult to define representative fields, Fig. 10 presents some examples.

## Discussion

### The burden of thyroid cancer and the limitations of cytology

In the last decades, the incidence of thyroid cancer has more than tripled [14]. Following this tendancy, in 2019, papillary thyroid cancer would become be the third most prevalent cancer among women in the United States, with associated annual costs estimated between 19 and 21 billion dollars [15]. This increase is mainly due to “small papillary carcinomas”, and is probably attributable to increased sreening and the more widespread use of ultrasound imaging [16].

As thyroid cancer is an indolent disease, the typical clinical presentation is that of an incidentally detected thyroid nodule, either on physical or radiologic examination [2]. However, only 7 to 15% of thyroid nodules will prove to be malignant [2]. The diagnostic workup of these nodules is largely limited by the poor performance of cytology in providing a definitive diagnosis in a high percentage of cases.

The *Bethesda Cytology Classification*, recently updated [3], enables researchers, pathologists, and clinicians to use a common language when it comes to thyroid cytopathology. While the malignancy risks related to the *benign* and *malignant* categories are clear, categories of “unknown significance” represent a clinical dilema to the physician.

Commercialized biomarker assays, designed with the intent of clarifying the risk of malignancy for these cases, are both limited in their sensitivity or specificity and expensive. For example, the PCR mutational analysis of 7 genes commercialised under the name *ThyroSeq* (CBLPath, Rye Brook, NY) has a specificity of 99%, but its sensivity varies from 44 to 100% [2], depending on studies. The galactine-3 immunohistochemistry technique published by Bartolazzi et al. also has a high specificity (93%), but a lower sensitivity (78%) [2]. The 167 gene expression classifier described by Alexander et al., commercialized under the name *Afirma* (Veracyte, San Francisco, CA), has a 96% sensitivity, but a 31% specificity [17]. Finally, combination of the *ThyGenX* and *ThyraMir* tests (Interspace Diagnostics, Parsippany, NJ) has a sensitivity of 89% and a specificity of 85% [18].

### PACE4 as a biomarker

The family of proprotein convertases is a group of nine enzymes, composed of PC1/3, PC2, furin, PC4, PC 5/6, PACE4, PC7, SKI-1/S1P, and PCSK9. Enzymes from this family, especially furin and PACE4, have been investigated regarding their implications in tumor progression, since they are involved in the processing and activation of various protein precursors that have been associated with cancer progression, including tumor growth factor beta (TGFβ), matrix metalloproteinases (MMP), and insulin-like growth factor receptors (IGF1 R) [7].

It has been demonstrated that, among various cell lineages, overexpression of PACE4 confers oncogenic growth-sustaining functions [19, 20]. The central role of PACE4 in the sustained growth capabilities of prostate cancer cells has been demonstrated by the induction of proliferation arrest using either a PACE4 inhibitor or PACE4-specific gene-silencing tools [21]. Similar results have been obtained for breast cancer [12]. In recent work, Couture et al. characterized a novel PACE4 isoform, PACE4-altCT, that is generated by an alternative splicing mechanism, and is oncogenic in prostate cancer cells [13]. Also included in this study was a preliminary scan of numerous cancer tissues at the mRNA level to examine PACE4-FL and PACE4-altCT, which suggested that changes in splicing ratios could be indicative of oncogenic status.

PACE4 expression is known to be inconsistent among tissues. The *Human Protein Atlas* [22] and another previous study [23] suggested that expression levels in thyroid tissues are relatively low. To our knowledge, no study had focused until now on the expression pattern of PACE4 among different thyroid nodule types. Moreover, no previous study had investigated the full-length and alternative isoforms of PACE4 in thyroid tissues.

### PACE4-FL: A potential biomarker for malignancy

A good biomarker would need to be consistently expressed in malignant nodules, and absent of both non-lesional (normal) parenchyma and benign thyroid nodules. Our data suggest that PACE4-FL is not expressed in non-lesional thyroid parenchyma and in benign nodules and thus, has good specificity (95.8%; 95% CI: 78.8–99.9%) for malignancy. Hence, presence of this isoform could be a good “rule in” test for cancer.

On the other hand, the limited sensitivity of PACE4-FL for malignancy (52.9%; 95% CI, 27.81% to 77.02%) is mostly attributable to false negative results consistently obtained with the follicular variant of papillary carcinoma. Indeed, all of the follicular variant of papillary carcinoma samples and two out of five follicular carcinoma samples presented a low immunostaining. The high proportion of follicular malignancies selected in our study, due to its design, in comparison with its lower incidence in real-life cohorts, explain the low sensitivity of PACE4-FL that we report.

It is well established that in cytopathology, “follicular-type” lesions are the most difficult to evaluate. This difficulty is also encountered with some mutational panel biomarker assays. For example, in a multi-institutional, double-blinded study evaluating the performance of the molecular analysis of 17 oncogenic alterations in nodules, Beaudenon-Huibregtse et al. reported that 8 of the 14 false-negative results proved to be follicular variants of papillary carcinomas on final pathology [24]. While 4 of these were noninvasive and encapsulated, and thus would nowadays correspond to benign nodules of the NIFTP category, the other 4 showed invasive characteristics, and thus would still be considered malignant with updated criteria [24]. Nikiforov et al., in their prospective mutational panel analysis of 1 056 fine needle aspiration samples, reported that 13% of their false-negative results were follicular carcinomas on final pathology, and 62% were noninvasive encapsulated follicular variants of papillary carcinoma, which would be considered benign nowadays [25].

Two hypothesis can be proposed to explain false-negative results for follicular variants of papillary carcinomas. First, genetic mutations from one subtype of thyroid cancer to the other are quite variable and characteristic [26]. For example, the *BRAF V600E* mutation is strongly associated with conventional papillary carcinoma, *BRAF K601E*, with the follicular variant of papillary carcinoma, and *PTEN*, with follicular carcinoma [26]. As such, it is possible that PACE4-FL is in fact strongly correlated to the classical variant of papillary carcinoma, but less so with “follicular-type” malignancies. Second, it is well described that some mutations are strongly correlated with the aggressiveness or other specific clinical features of the thyroid cancer. For example, the *PAX8/PPARγ* rearrangement is linked, among others, to vascular invasion [27], while the *p53* mutation is linked to tumor dedifferentiation [26]. While the limited number of specimens included in this study precludes us to do so, further studies could explore if PACE4 is linked to a specific phenotype or clinical feature of thyroid cancer, and thus would rather be a marker of this phenotype. This might also be one path of explanation as to why the expression was varying among some nodule types, as for the medullar carcinomas.

Finally, as to the histochemical distribution, it is interesting to mention that the apical positivity of PACE4-FL, particularly marked for papillary carcinoma (Fig. 5a), is consistent with data by Couture et al. [13] and Nour et al. [28], suggesting that PACE4-FL is readily reaching the cell surface and accumulating in the extracellular matrix.

### PACE4-altCT: A demonstration of the relationship between PACE4-FL and PACE4-altCT in alternative splicing

As opposed to PACE4-FL, PACE4-altCT did not prove to be either sensitive nor specific for malignancy. However, while PACE4-FL is more expressed in cancer than in adjacent normal parenchyma, the opposite is true for PACE4-altCT: a statistically significant reduction in PACE4-altCT was noted in malignant nodules when compared to adjacent non-lesional parenchyma (Fig. 7).

As such, the present study on PACE4 expression in thyroid cancer presents a contrast with data obtained in previous work on prostate cancer. In prostate cancer, there is a clear shift in alternative splicing to favour PACE-altCT [13], while in the present study, the inverse appears to be true. While our data support the tight relationship between these two isoforms, it also suggests that in thyroid carcinoma, different mechanisms are affecting the alternative splicing events. In prostate cancer, increased PACE4-altCT was shown to be due to hypomethylating epigenetic events that favour PACE4-altCT formation [13]. Although this was not verified in the present study, it is possible that increased DNA methylation is occurring in thyroid cancer, leading to alternative splicing events that favour PACE4-FL. Indeed, DNA methylation signatures have been examined and shown to have specific patterns in various thyroid subtypes [29]. However, since our study only focussed on PACE4 isoforms analysis in terms of proteins without examining RNA levels nor splicing activities, further studies will be needed to determine if PACE4-FL increases are due to gene overexpression or splicing regulation. Correlation with methylation signatures would also provide a complete picture of how PACE4 is involved in thyroid carcinoma, as previously done for other cancer types [13].

### Limitations of the study

- The number of samples per thyroid pathology is limited, although statistical significance was obtained. A larger sampling would be of interest and may allow to identify additional statistically significant differences between the various pathologies, owing to a higher study power.
- Immunohistochemistry is a semi-quantitative method, with inherent interobserver variability and subjectivity. While quantitative results could have been obtained with immunofluorescence, no immunofluorescence method have been described as of today for PACE4.

### Further studies

A follow-up study, using quantitative Polymerase Chain Reaction (qPCR) to determine the quantitative expression levels as well as the splicing indexes of PACE4 among thyroid cytology specimens, is currently being designed to determine the clinical usefulness of PACE4 as a biomarker. Studies to correlate methylation signature and PACE4 alternative splicing in thyroid cancer subtypes would also be highly informative. Further studies could also investigate the yield of a combination of PACE4-FL to other described biomarkers more specific for follicular lesion. Finally, as there is currently a lack of targeted therapy in thyroid cancer, especially for non-iodine-avid lesions [26], and as an inhibitor of PACE4 has already been developed and tested *in cellulo* for various cancer types [30], PACE4 could eventually be explored as an oncologic target in thyroid cancer.

## Conclusion

This study is the first of its kind to explore the expression of PACE4, in both its full-length and alternative isoforms, in lesional and non-lesional thyroid parenchyma, using previously validated antibodies and an automatized immunohistochemistry technique. Our results suggest that PACE4-FL is not constitutionally expressed in normal thyroid tissue, and that its expression is highly specific to malignancy, making it a potential “rule in” test for cancer. PACE4-altCT displayed the opposite relationship, being less expressed in malignant thyroid nodules than in non-lesional thyroid parenchyma. This relationship is consistent with the alternative splicing mechanism of PACE4 and suggests that increased methylation may be occurring in thyroid cancer. This study justifies further research on the subject, with the intent of defining more precisely the role of PACE4 as a biomarker for thyroid malignancy and as a potential oncologic target.

## Additional files

Additional file 1: Original data spreadsheet. Contains the raw data, used for statistical analysis. (XLSX 50 kb)

Additional file 2: Multiple comparison analyses of the percentage of high immunostaining for PACE4-FL and PACE4-altCT among benign versus malignant nodules. *p* values were calculated with the Fischer exact test, and adjusted with the false discovery rate (FDR) correction. * Comparisons for which exact Fisher test could not be computed. (PPTX 42 kb)

## Abbreviations

FN/SFN: Follicular neoplasm – Suspicion of follicular neoplasm 621
IGF1 R: Insulin-like Growth Factor One Receptor 626
MMP: Matrix Metalloproteinases 625
NIFTP: Noninvasive Follicular Thyroid Neoplasm with Papillary-like Nuclear Features
qPCR: Quantitative Polymerase Chain Reaction 623
SUSP: Suspicious for malignancy 622
TGFβ: Tumor Growth Factor Beta 624
US/FLUS: Atypia of unknown significance – Follicular lesion of unknown significance 620
