# Supplementary material for: Evaluation of PACE4 isoforms as biomarkers in thyroid cancer
Source: J Otolaryngol Head Neck Surg. 2018 Oct 19;47:63. doi: 10.1186/s40463-018-0311-x (PMC6194618; doi:10.1186/s40463-018-0311-x)
Supplement: Supplementary file 2 — Multiple comparison analyses of the percentage of high immunostaining for PACE4-FL and PACE4-altCT among benign versus malignant nodules. p values were calculated with the Fischer exact test, and adjusted with the false discovery rate (FDR) correction. * Comparisons for which exact Fisher test could not be computed. (PPTX 42 kb) [file 40463_2018_311_MOESM2_ESM.pptx]

## Slide 1
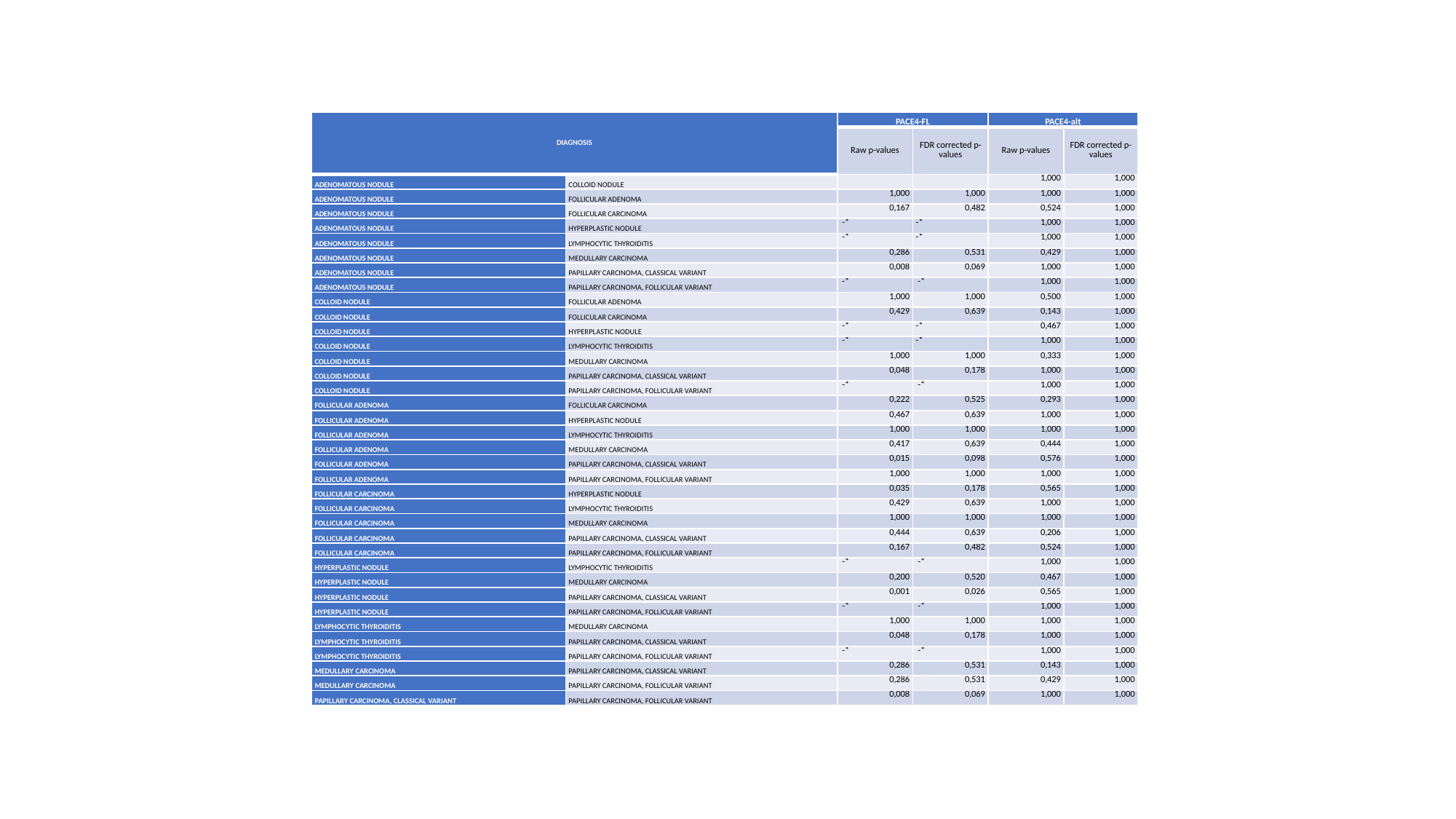

| DIAGNOSIS | | PACE4-FL | | PACE4-alt | |
| --- | --- | --- | --- | --- | --- |
| | | Raw p-values | FDR corrected p-values | Raw p-values | FDR corrected p-values |
| ADENOMATOUS NODULE | COLLOID NODULE | | | 1,000 | 1,000 |
| ADENOMATOUS NODULE | FOLLICULAR ADENOMA | 1,000 | 1,000 | 1,000 | 1,000 |
| ADENOMATOUS NODULE | FOLLICULAR CARCINOMA | 0,167 | 0,482 | 0,524 | 1,000 |
| ADENOMATOUS NODULE | HYPERPLASTIC NODULE | -\* | -\* | 1,000 | 1,000 |
| ADENOMATOUS NODULE | LYMPHOCYTIC THYROIDITIS | -\* | -\* | 1,000 | 1,000 |
| ADENOMATOUS NODULE | MEDULLARY CARCINOMA | 0,286 | 0,531 | 0,429 | 1,000 |
| ADENOMATOUS NODULE | PAPILLARY CARCINOMA, CLASSICAL VARIANT | 0,008 | 0,069 | 1,000 | 1,000 |
| ADENOMATOUS NODULE | PAPILLARY CARCINOMA, FOLLICULAR VARIANT | -\* | -\* | 1,000 | 1,000 |
| COLLOID NODULE | FOLLICULAR ADENOMA | 1,000 | 1,000 | 0,500 | 1,000 |
| COLLOID NODULE | FOLLICULAR CARCINOMA | 0,429 | 0,639 | 0,143 | 1,000 |
| COLLOID NODULE | HYPERPLASTIC NODULE | -\* | -\* | 0,467 | 1,000 |
| COLLOID NODULE | LYMPHOCYTIC THYROIDITIS | -\* | -\* | 1,000 | 1,000 |
| COLLOID NODULE | MEDULLARY CARCINOMA | 1,000 | 1,000 | 0,333 | 1,000 |
| COLLOID NODULE | PAPILLARY CARCINOMA, CLASSICAL VARIANT | 0,048 | 0,178 | 1,000 | 1,000 |
| COLLOID NODULE | PAPILLARY CARCINOMA, FOLLICULAR VARIANT | -\* | -\* | 1,000 | 1,000 |
| FOLLICULAR ADENOMA | FOLLICULAR CARCINOMA | 0,222 | 0,525 | 0,293 | 1,000 |
| FOLLICULAR ADENOMA | HYPERPLASTIC NODULE | 0,467 | 0,639 | 1,000 | 1,000 |
| FOLLICULAR ADENOMA | LYMPHOCYTIC THYROIDITIS | 1,000 | 1,000 | 1,000 | 1,000 |
| FOLLICULAR ADENOMA | MEDULLARY CARCINOMA | 0,417 | 0,639 | 0,444 | 1,000 |
| FOLLICULAR ADENOMA | PAPILLARY CARCINOMA, CLASSICAL VARIANT | 0,015 | 0,098 | 0,576 | 1,000 |
| FOLLICULAR ADENOMA | PAPILLARY CARCINOMA, FOLLICULAR VARIANT | 1,000 | 1,000 | 1,000 | 1,000 |
| FOLLICULAR CARCINOMA | HYPERPLASTIC NODULE | 0,035 | 0,178 | 0,565 | 1,000 |
| FOLLICULAR CARCINOMA | LYMPHOCYTIC THYROIDITIS | 0,429 | 0,639 | 1,000 | 1,000 |
| FOLLICULAR CARCINOMA | MEDULLARY CARCINOMA | 1,000 | 1,000 | 1,000 | 1,000 |
| FOLLICULAR CARCINOMA | PAPILLARY CARCINOMA, CLASSICAL VARIANT | 0,444 | 0,639 | 0,206 | 1,000 |
| FOLLICULAR CARCINOMA | PAPILLARY CARCINOMA, FOLLICULAR VARIANT | 0,167 | 0,482 | 0,524 | 1,000 |
| HYPERPLASTIC NODULE | LYMPHOCYTIC THYROIDITIS | -\* | -\* | 1,000 | 1,000 |
| HYPERPLASTIC NODULE | MEDULLARY CARCINOMA | 0,200 | 0,520 | 0,467 | 1,000 |
| HYPERPLASTIC NODULE | PAPILLARY CARCINOMA, CLASSICAL VARIANT | 0,001 | 0,026 | 0,565 | 1,000 |
| HYPERPLASTIC NODULE | PAPILLARY CARCINOMA, FOLLICULAR VARIANT | -\* | -\* | 1,000 | 1,000 |
| LYMPHOCYTIC THYROIDITIS | MEDULLARY CARCINOMA | 1,000 | 1,000 | 1,000 | 1,000 |
| LYMPHOCYTIC THYROIDITIS | PAPILLARY CARCINOMA, CLASSICAL VARIANT | 0,048 | 0,178 | 1,000 | 1,000 |
| LYMPHOCYTIC THYROIDITIS | PAPILLARY CARCINOMA, FOLLICULAR VARIANT | -\* | -\* | 1,000 | 1,000 |
| MEDULLARY CARCINOMA | PAPILLARY CARCINOMA, CLASSICAL VARIANT | 0,286 | 0,531 | 0,143 | 1,000 |
| MEDULLARY CARCINOMA | PAPILLARY CARCINOMA, FOLLICULAR VARIANT | 0,286 | 0,531 | 0,429 | 1,000 |
| PAPILLARY CARCINOMA, CLASSICAL VARIANT | PAPILLARY CARCINOMA, FOLLICULAR VARIANT | 0,008 | 0,069 | 1,000 | 1,000 |
